# Utilizing Closed Incisional Negative Pressure Therapy Reduces Peripheral Bypass Infection Rates Without Increasing Costs

**DOI:** 10.7759/cureus.9217

**Published:** 2020-07-16

**Authors:** Jacob J Frisbie, Stefano J Bordoli, Justin M Simmons, Sandra K Zuiderveen

**Affiliations:** 1 Vascular Surgery, Spectrum Health, Grand Rapids, USA; 2 Vascular Surgery, Spectrum Health, Grand Rpaids, USA; 3 Quality Improvement, Spectrum Health, Grand Rapids, USA

**Keywords:** peripheral arterial diseases, re-vascularization, peripheral vascular surgery, closed incision negative pressure wound therapy, infection control measures, infection, infection prevention and control

## Abstract

Introduction: We evaluated outcomes of closed incisional negative pressure therapy (ciNPT) on surgical site infection (SSI) rates in lower extremity bypass patients. We sought to determine whether or not the routine use of ciNPT is a cost-effective measure.

Methods: During a period from May 2018 to August 2018, our institution transitioned to the routine use of ciNPT for re-vascularization procedures. We retrospectively reviewed our outcomes before and after the initiation of ciNPT. Group A included patients from September 2017 to April 2018 without ciNPT and Group B included patients from September 2018 to April 2019 with ciNPT. Chi-squared analysis was performed and the p value was set at <0.05 to obtain statistical significance. Cost analysis was separately performed utilizing hospital metrics.

Results: There were a total of 102 patients in Group A and 113 patients in Group B. There was no difference in demographic information between the two groups. The overall SSI rate for Group A was 11.8% (12/102). Group B had an overall SSI rate of 3.5% (4/113; p=0.02). Deep infection rate for Group A was 7% (7/102) and for Group B was 1% (1/113; p=0.01). Cost analysis demonstrated a minimum of $62,000 in infection-related cost savings between both groups.

Conclusions: ciNPT has had a profound effect on our practice and has resulted in a decrease in both deep and superficial infections. This has led to a significant cost-effective measure for our institution. We now routinely use ciNPT on all lower extremity bypass patients.

## Introduction

Around 10%-20% of patients undergoing lower extremity re-vascularization experience a surgical site infection (SSI) [1]. In the United States, SSIs increase healthcare spending by approximately $1.6 billion per year [2]. Many factors contribute to the high infection rates of extremity bypass procedures. Proximity of femoral incisions to genitalia and inguinal lymphatics can make these incisions problematic [1]. This area can be extremely difficult to keep clean and dry.

The use of closed incisional negative pressure therapy (ciNPT) has been shown to decrease SSIs for inguinal incisions [3-5]. It has also been shown to be cost effective in high-risk patients [6,7]. We sought to identify if the routine use of ciNPT is cost effective for use in all extremity bypass patients. The routine use of ciNPT was undertaken in extremity bypass patients at our institution. We performed a retrospective chart review on patients both before and after the institution of routine ciNPT.

## Materials and methods

We conducted a retrospective chart review on patients who had a surgical bypass procedure before and after the routine use of ciNPT (Table 1). Institutional Review Board approval was obtained to conduct our retrospective chart review. This time frame was chosen as our institution has now transitioned to the routine use of ciNPT on all bypass patients. Our institution transitioned to the routine use of ciNPT because of a prior surge in bypass infection rates. All procedures were performed at a single institution in a group of eight vascular surgeons. A four-month overlap period (May 2018 to August 2018) was allowed for physicians to become comfortable with the device and familiarize nurses with the product. This overlap period was not included in the study. All ciNPT procedures were performed utilizing Prevena™ (3M + KCI, St. Paul, MN). All patients were instructed by a clinical nurse upon discharge to maintain the dressing for a duration of seven days. All devices were placed utilizing standard settings. The Prevena device does not allow adjustment of pressure. Proximal groin incisions received ciNPT and distal incisions received a standard dressing. Inclusion criteria included having a re-vascularization procedure performed during Group A and B periods. One patient in each group was excluded from the study due to patient expiration before 90 days. Both cases were unrelated to infection and incisions were healing as expected per chart review.

**Table 1:** Group divisions

Group A	Group B
September 2017-April 2018	September 2018-April 2019
Before the introduction of negative pressure wound incisional therapy	Routine use of negative pressure wound incisional therapy
102 patients	113 patients

Demographic variables were compared between Group A and Group B. Demographic variables studied include gender, presence of diabetes, current smoking status, hyperlipidemia, use of prosthetic graft, femoral incision and presence of redo operative field. Primary outcomes included deep and overall infection rates. Secondary outcomes included cost savings. We hypothesized that after the routine use of ciNPT, our deep and overall infection rates would decrease and thus result in cost savings. Testing was done with chi-square or Fisher's exact test depending on cell count to determine if there was an association between the groups and the variables tested. Continuous variables were tested using a Kruskal-Wallis test. Statistical significance was set at a level of 0.05. Categorical variables are displayed as counts and percentages while continuous data is displayed as mean with the inter-quartile range (IQR). Determination of infection was based on National Healthcare Safety Network (NHSN) classification guidelines. Superficial infections did not violate fascia. Deep infections had sub-fascial involvement. Graft infections included the bypass graft. Patients underwent surveillance for 90 days to determine if they had a superficial or deep infection. Patients with re-vascularization procedures were seen in the vascular office within one month of hospital discharge. Pictures of the wounds were taken if there were any concerning features. If patients were not seen in the office, they were called by a clinical nurse and again at 90 days. Patients were followed up for one year to determine if they had a graft infection. This follow-up was performed by the vascular quality department utilizing phone calls and scheduling a visit if a problem was present. Cost analysis was performed utilizing internal cost metrics at our institution.

## Results

There was no difference between Group A or Group B in terms of demographical information including gender, presence of diabetes, current smoking status, hyperlipidemia, whether a vein bypass or prosthetic bypass (polytetrafluethylene [PTFE] or Dacron® [Invista, Wichita, KS]) was performed, presence of femoral incision, and if the procedure was performed in a redo operative field (Table 2).

**Table 2:** Demographics of patients *Bovine carotid or cadaveric graft.

	All	Group A	Group B	p value
Total (N)	215	102	113	
Gender				0.340
Female	56 (26.0%)	23 (22.5%)	33 (29.2%)	
Male	159 (74.0%)	79 (77.5%)	80 (70.8%)	
BMI	27.8 [24.6; 31.4]	28.2 [25.1; 31.5]	27.6 [24.2; 31.2]	0.263
Diabetes mellitus				0.763
No	134 (62.3%)	62 (60.8%)	72 (63.7%)	
Yes	81 (37.7%)	40 (39.2%)	41 (36.3%)	
Current smoker				0.730
No	108 (50.2%)	53 (52.0%)	55 (48.7%)	
Yes	107 (49.8%)	49 (48.0%)	58 (51.3%)	
Hyperlipidemia				0.201
No	15 (6.98%)	10 (9.80%)	5 (4.42%)	
Yes	200 (93.0%)	92 (90.2%)	108 (95.6%)	
Vein graft				0.177
No	121 (56.3%)	52 (51.0%)	69 (61.1%)	
Yes	94 (43.7%)	50 (49.0%)	44 (38.9%)	
Prosthetic				0.110
No	101 (47.2%)	54 (53.5%)	47 (41.6%)	
Yes	113 (52.8%)	47 (46.5%)	66 (58.4%)	
Other*				0.482
No	207 (96.3%)	97 (95.1%)	110 (97.3%)	
Yes	8 (3.72%)	5 (4.90%)	3 (2.65%)	
Non-femoral procedure				0.57
No	199 (92.6%)	96 (94.1%)	103 (91.2%)	
Yes	16 (7.4%)	6 (5.9%)	10 (8.8%)	
Redo procedure				0.238
No	153 (71.2%)	77 (75.5%)	76 (67.3%)	
Yes	62 (28.8%)	25 (24.5%)	37 (32.7)	

The overall SSI rate for Group A was 11.8% (12/102). Group B had an overall SSI rate of 3.5% (4/113). Deep infection rate for Group A was 7% (7/102) and that for Group B was 1% (1/113) (Table 3).

**Table 3:** Deep and overall infection rates

	Group A - actual	Group B - actual	Group A - expected	Group B - expected	Chi-square	p value
Deep Infection	7	1	3.7	4.3	12.07	0.016867
Overall infection	12	4	7.59	8.41	11.47	0.021758
Total patients	102	113				

Cost analysis demonstrated a minimum of $62,000 in infection-related cost savings between Group A and Group B even when accounting for the total cost of negative pressure wound therapy application (Table 4).

**Table 4:** Cost analysis for Groups A and B SSI, surgical site infection; VAC, vacuum-assisted closure. Deep SSI encountered in Group B is the actual hospital cost for patient. Approximate cost superficial SSI = $5,000. Approximate cost deep SSI = $20,000. Approximate cost graft infection = N/A. Approximate cost of Prevena = $495.

	Group A	Group B
Cost superficial SSI	$25,000	$15,000
Cost deep SSI	$140,000	$32,000 (actual)
Cost graft infection	$0	$0
Total infection cost	$165,000	$47,000
Total cost incisional VAC	$0	$55,935
Total cost	$165,000	$103,000

## Discussion

SSIs are a significant burden for patients as well as have an impact on the healthcare expenditure [2]. Superficial infections may only require outpatient treatment; however, some patients present to the Emergency Department and require IV antibiotics. Deep infections typically require IV antibiotics as well as surgical incision and drainage. Graft infections require an extensive hospital stay with graft explanation, IV antibiotics, and in most cases re-vascularization.

There are limitations to our study. First and foremost, this is a retrospective, single-center study. There have been randomized controlled trials demonstrating that negative pressure wound therapy decreases SSIs in vascular patients [6-8]. However, no randomized controlled trials have investigated routine use and cost savings in peripheral bypass patients. Secondly, our study was unable to account for malfunctioning Prevena devices or the length of time that they were maintained. Because bypass patients are typically discharged from the hospital within seven days, we have no way of telling if all patients complied with maintaining their dressing for a full seven days. In addition, pre-operative bundles were instituted during the time period of Group B. In January 2019, a SSI bundle was initiated at our institution. This included chlorohexidine bath and nasal swabs pre-operatively. Peri-operative antibiotic dosing was also tracked much more closely during the time period of Group B. The initiation of surgical bundles has been associated with reduced infection rates, although the literature has shown that ciNPT can decrease infection rates independently of SSI prevention bundles [4,9]. This is a cofounding variable but perhaps ciNPT should be included in surgical bundles if reduced infections and cost savings are the result.

It is difficult to estimate costs associated with infections. Utilizing internal data for cost analysis is not ideal but offers the greatest insight for a hospital system. The cost of SSIs varies widely from institution to institution. Our cost of deep infections at $20,000 is a very low estimation. Cost savings between the two groups was likely much more significant than our cost analysis conveys. Deep infections often require multiple surgical debridements as well as an extended hospital stay. Moreover, vascular surgery patients typically have multiple co-morbid conditions that contribute to the observed infection rate of 10%-20% in re-vascularization procedures [1]. In fact, the American Society of Anesthesiologists (ASA) categorizes re-vascularization as a "high risk" procedure. The selective use of ciNPT may save our institution money if it is withheld in healthy patients. However, these patients are rare in the vascular world. If ciNPT were to prevent two deep infections per hundred bypasses, the device will pay for itself and prevent patient morbidity.

## Conclusions

Routine use of ciNPT is cost preventative and decreases both deep and superficial SSIs in vascular bypass patients. Our group has transitioned to the routine use of Prevena incisional therapy in all bypass patients as it has proven to be a cost-effective measure.
